# Effect of difference in occlusal contact area of mandibular free-end edentulous area implants on periodontal mechanosensitive threshold of adjacent premolars

**DOI:** 10.1186/s40064-015-1497-2

**Published:** 2015-11-17

**Authors:** Rie Terauchi, Korenori Arai, Masahiro Tanaka, Takayoshi Kawazoe, Shunsuke Baba

**Affiliations:** Department of Oral Implantology, Osaka Dental University, Hirakata, Japan; Department of Fixed Prosthodontics and Occlusion, Osaka Dental University, Hirakata, Japan

**Keywords:** Implant, Occlusal contact, Periodontal mechanosensitive threshold

## Abstract

Implant treatment is believed to cause minimal invasion of remaining teeth. However, few studies have examined teeth adjacent to an implant region. Therefore, this study investigated the effect of occlusal contact size of implants on the periodontal mechanosensitive threshold of adjacent premolars. A cross-sectional study design was adopted. The Department of Oral Implantology, Osaka Dental University, was the setting where patients underwent implant treatment in the mandibular free-end edentulous area. The study population comprised of 87 patients (109 teeth) who underwent follow-up observation for at least 3 years following implant superstructure placement. As variables, age, sex, duration following superstructure placement, presence or absence of dental pulp, occlusal contact area, and periodontal mechanosensitive threshold were considered. The occlusal contact area was measured using Blue Silicone^®^and Bite Eye BE-I^®^. Periodontal mechanosensitive threshold were measured using von Frey hair. As quantitative variables for periodontal mechanosensitive threshold, we divided subjects into two groups: normal (≤5 g) and high (≥5.1 g). For statistical analysis, we compared the two groups for the sensation thresholds using the Chi square test for categorical data and the Mann–Whitney U test for continuous volume data. For variables in which a significant difference was noted, we calculated the odds ratio (95 % confidence interval) and the effective dose. There were 93 teeth in the normal group and 16 teeth in the high group based on periodontal mechanosensitive threshold. Comparison of the two groups indicated no significant differences associated with age, sex, duration following superstructure placement, or presence or absence of dental pulp. A significant difference was noted with regard to occlusal contact area, with several high group subjects belonging to the small contact group (odds ratio: 4.75 [1.42–15.87]; effective dose: 0.29). The results of this study suggest an association between implant occlusal contact area and the periodontal mechanosensitive threshold of adjacent premolars. Smaller occlusal contact application resulted in an increased threshold. It appears that prosthodontic treatment should aim not only to improve occlusal function but also to maintain oromandibular function with regard to the preservation of remaining teeth.

## Background

In the recent years, implant treatment has been widely used for prosthodontic treatment of edentulous areas because it offers high success rates and good long-term postoperative outcomes. Moreover, there is lesser invasion of the remaining teeth than in other types of prosthodontic treatment. This reduces the burden placed on the remaining teeth and should therefore facilitate their preservation. However, few reports have investigated the long-term prognosis of the remaining natural teeth (Yamazaki et al. 2013). Further, there have been few studies elucidating the optimal size of the occlusal contact to be applied in the implant superstructure, and no clear concept has been outlined regarding the same. In the clinical setting, to compensate for the difference between pressure displacement of implants and natural teeth, there is a tendency to apply lesser occlusion in implants than in natural teeth (Kim et al. 2005). However, no studies have yet analyzed the clinical efficacy of occlusal contact application in this concept.

The periodontal membrane is a dense connective tissue between the tooth and alveolar bone. The periodontal membrane not only functions as a support and fixation device for teeth but also receives rich sensory innervation and functions as an important sensory device for the oral cavity. Sensations in the periodontal membrane that include sense of pain, contact, pressure, tooth location, and proprioception (deep sensation) are received by periodontal mechanoreceptors. Information perceived by these receptors is directed to the central nervous system through various oral reflexes for the nervous control of mastication. Furthermore, this is associated with information related to food size and hardness (Inoue et al. 1989) and enhances taste sensation. There have been previous studies reporting the tactile and pressure sensitivity thresholds in periodontal membranes of natural teeth and the measurement of sensory capabilities in implants (Yamauchi 1988; Habre-Hallage et al. 2010; Grieznis et al. 2010). However, there have been no studies examining the periodontal mechanosensitive threshold of the remaining natural teeth in implant patients.

Therefore, in this study, we analyzed the occlusal contact area in the implant region against the periodontal mechanosensitive threshold in teeth adjacent to the implant region, and we investigated the influence of implant treatment on the remaining teeth.

## Methods

### Study design

This was a cross-sectional study, involving a survey conducted between April 2014 and March 2015 among patients who had undergone implant treatment in the mandibular free-end edentulous area at the department of oral implantology, Osaka Dental University. During this study, the revised version of the Declaration of Helsinki (Edinburgh edition; October 2000) was respected. A research protocol that considered subjects’ human rights and the protection of their benefits was drawn up and approved by the ethical review board of Osaka Dental University (approval number: 110782).

### Participants

Patients who had progressed well for at least 3 years following superstructure placement as part of the implant treatment of the mandibular free-end edentulous area and had provided their informed consent after receiving an explanation regarding the outline of this study were included in this study. Exclusion criteria were abnormalities in the stomatognathic system and a history of orthodontic treatment. If there was an edentulous area at any other site in the oral cavity, the patients were included in the study if they had undergone bridge or implant treatment, and excluded if they had been treated with removable partial dentures. The subject teeth were implant-adjacent premolars with occlusal contact and no periodontal disease (pocket probing depth: 3 mm or less; no bleeding on probing) (Japanese Society of Periodontology 2009).

### Study size

This epidemiological study aimed to understand the prevalent state of medical treatment by analyzing all registered patients to ensure maximum validity of the results obtained.

### Variables investigated

The variables investigated in this study were age, sex, period following superstructure placement, presence or absence of dental pulp, periodontal mechanosensitive threshold of adjacent teeth, and occlusal contact area.

### Data sources and measurement methods

#### Age, sex, and period following superstructure placement

These were extracted from the medical records and the presence or absence of dental pulp was determined using radiographic images.

#### Periodontal membrane tactile and pressure sensitivity thresholds

Periodontal mechanosensitive threshold were measured using von Frey hair (Aesthesio^®^; DanMic Global, California, USA). The subjects were seated with their head in contact with the dental unit’s headrest and their occlusal plane parallel to the floor. An Angle Wider was used to retract the lips and surgical tape (Micropore; 3M, Tokyo, Japan) was affixed to the tooth surface in the stimulation area. Subjects were requested to shut their eyes, the tip of the von Frey hair was brought in contact with the target tooth, and measurements were recorded. The stimulation was directed perpendicular to the long axis of the tooth, from the tooth crown buccal surface towards the lingual surface. The stimulated area was the mesiodistal center of the occlusal third of the crown.

**Fig. 1:**
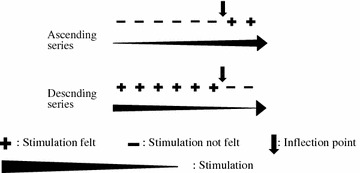
Psychophysical assessment (up–down method)

Periodontal membrane tactile and pressure sensitivity thresholds of the adjacent teeth were determined using the up–down method of psychophysical assessment (Fig. 1). Stimulation was initiated with a small, imperceptible stimulus, after which the intensity was increased. The stimulation was repeated until the subject could sense it, after which the stimulation was stopped. During this period, the inflection point was measured as the median value between the minimum level at which the subject could sense the stimulation and the maximum level at which the subject could not sense the stimulation, and the values were arranged in an ascending series. The stimulation was then started in a reverse order from when the stimulus could be sensed and was repeatedly applied with decreasing intensity until the subject could no longer sense it, after which the stimulation was stopped. Here the inflection point was measured as the median value between the minimum level at which the subject could not sense the stimulation and the maximum level at which the subject could sense the stimulation, and the values were arranged in a descending series. The ascending and descending series were each repeated twice. When the subject’s reaction did not stabilize, measurements were performed until they did and measurement values from two stable cycles were used. The median value of the inflection points from the ascending and descending series were used as periodontal membrane tactile and pressure sensitivity thresholds.

#### Occlusal contact area measurement

Occlusal contact area was measured by registering a check-bite in the intercuspal position using silicone impression materials (Blue Silicone^®^; GC, Tokyo, Japan). A tooth-contact analyzing device (Bite Eye^®^; GC, Tokyo, Japan) was used to take measurements after setting thresholds such that occlusal contact thickness was ≤30 μm.

### Quantitative variables

Based on the periodontal mechanosensitive threshold, the subjects were divided into two groups: normal and high. Periodontal membrane tactile and pressure sensitivity thresholds were set at ≤5 g for the normal group and ≥5.1 g for the high group, based on Mukai et al. (2011) reported that interquartile range of periodontal mechanosensitive threshold of mandibular premolar teeth of adult with natural dentition was less than 5 g. Clinically, there is a tendency to apply a smaller amount of occlusal contact area to implants than that found in the remaining teeth (Misch and Bidez 1994). However, in the dentition of healthy-toothed individuals, occlusal contact area of distal natural teeth tends to be large (Araki et al. 1997). Therefore, in this study, we divided subjects into two groups based on implant occlusal contact area: a small contact group, wherein implant occlusal contact area was less than half that of the adjacent premolars, and a large contact group, wherein implant occlusal contact area was at least half that of the adjacent premolars.

### Statistical analyses

The two periodontal mechanosensitive threshold groups were compared by testing categorical data with the Chi square test and continuous data with the Mann–Whitney U test. The odds ratio (95 % confidence interval) and effective dose were calculated for variables that exhibited significant differences. For categorical data in which a significant difference was noted, the periodontal mechanosensitive threshold were converted to continuous data and were examined using the Mann–Whitney U test. IBM SPSS Statistics Ver.22 (IBM Corporation, New York, USA) was used for statistical analysis.

## Results

### Subjects

The subjects in this study comprised 87 patients (men: 33, women: 54; mean age: 61.4 ± 7.7 years; subject teeth: 109) of the Department of Oral Implantology, Osaka Dental University.

### Comparison of the two periodontal mechanosensitive threshold groups

Based on the periodontal membrane tactile and pressure sensitivity thresholds, there were 93 teeth in the normal group and 16 teeth in the high group. Comparison of the two threshold groups indicated no statistically significant differences in relation to age, sex, period following superstructure placement, or presence or absence of dental pulp. A significant difference was noted with regard to occlusal surface area, with significantly high values noted in the small contact group. The odds ratio was 4.75 (1.42–15.87) and the effective dose was 0.29 (Table 1).

**Table 1:** Background data of the normal and high periodontal mechanosensitive threshold groups

	Periodontal mechanosensitive threshold				
	Normal group (n = 93)	High group (n = 16)	p value	Odds ratio (95 % CI)	Effective dose
Median age (range)	63.0 (39–86)	58.0 (49–77)	0.992		
Sex (n) (%)					
Male	35 (38)	6 (38)	0.549		
Female	58 (62)	10 (62)			
Period following superstructure placement (days)					
Median days (range)	1451.5 (1102–3595)	1520 (1108–3374)	0.844		
Implant occlusal contact area (n) (%)					
Small contact group	36 (39)	12 (75)	0.003	4.7 (1.4–15.9)	0.29
Large contact group	57 (61)	4 (25)			
Dental pulp in adjacent teeth (n) (%)					
Yes	51 (55)	8 (50)	0.306		
No	42 (45)	8 (50)			

Normal group: periodontal mechanosensitive threshold is ≤5 g. High group: periodontal mechanosensitive threshold is ≥5.1 g in implant-adjacent premolars. Small contact group: implant occlusal contact area is less than half that of adjacent premolars. Large contact group: implant occlusal contact area is at least half that of adjacent premolars. Categorical data were examined using the Chi square test and continuous data were examined using the Mann–Whitney U test

### Comparison of periodontal membrane tactile and pressure sensitivity threshold values between small and large contact groups

**Fig. 2:**
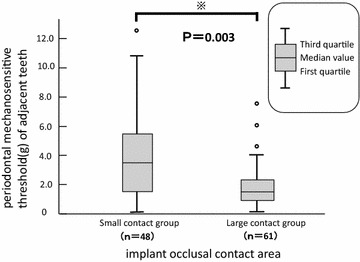
Comparison of periodontal mechanosensitive threshold in small and large contact groups. *Small contact group*: peri-implant occlusal contact area is less than half that of adjacent premolars. *Large contact group*: peri-implant occlusal contact area is at least half that of adjacent premolars

Because a significant difference was noted with regard to occlusal contact area, the sensitivity thresholds were converted into continuous data and investigated. In the small contact group, the threshold values ranged between 12.38 and 0.12 g, and the median value was 3.25 g. In the large contact group, the threshold values ranged between 7.50 and 0.12 g, and the median value was 1.45 g, indicating a statistically significant difference (p = 0.003; Fig. 2).

## Discussion

### Research methods

#### Measurement of periodontal mechanosensitive threshold

There are various methods for measuring periodontal mechanosensitive threshold, with reports of techniques using von Frey hair (Manly et al. 1952), spring esthesiometers (Loewenstein and Rathkamp 1955), and strain gauges (Keller et al. 1996; Hammerle et al. 1995). Von Frey hair is a test method wherein a hair-shaped filament is pushed against the target tooth until it bends and the intensity of the mechanical stimulation applied to the target tooth is varied based on the pre-set filament thickness. With regard to clinical application, we selected von Frey hair because it can be used for simple and quantitative measurement. To measure sensitivity thresholds, we used the up–down method of psychophysical assessment because it permits quantitative measurement.

#### Occlusal contact area measurement

Clinically, Blue Silicone^®^ was used to measure occlusal contact area and a tooth contact analyzing device was used for analysis. Blue Silicone^®^ is thin, with a minimum film thickness of 4 μm, and is appropriate for grasping occlusal contact state aspect which is a close-to-true occlusal contact state. The Bite Eye is considered to have extremely high reproducibility for repeated measurements (Uchida et al. 2014). We selected silicone with a thickness of ≤29 μm, which is close to the thickness of the occlusal registration paper (30 μm) that is often used in usual clinical settings.

Occlusal contact state is thought to be affected by the intensity of tooth clenching and it has been stipulated that it is optimal to measure muscular activity using electromyography (Mukai et al. 2011). However, in this study, occlusal contact area was studied not as a comparison between subjects, but as a ratio between the occlusal contact areas of the implant and adjacent premolars in each subject. Therefore, the tooth-clenching intensity was set at “strong clenching” and the magnitude was left to the perception of the subjects.

#### Target teeth

The molars directly receive mechanical stimulation during mastication, and molar occlusal state and occlusal force are strongly related to occlusal support. With regard to the relationship between mastication and molar region occlusal support, masticatory performance is considered to be high in individuals with a large occlusal contact area and high maximum occlusal force (Takehara and Honda 2000). In patients who have undergone implant treatment in the free-end edentulous implant area, occlusion is also thought to play an important role during mastication. Normally, it is considered that in the natural dentition, occlusal contact area increases from the mesial to the distal. However, currently, occlusal contact area of distally located implants is often set smaller than that of adjacent teeth. Therefore, we set target teeth in this study to investigate this discrepancy.

### Results

It has been reported that implant overload can cause bone resorption and inflammation (Isidor 1996; Miyata et al. 2000). Implant-protected occlusion proposed by Misch and Bidez (1994) aims to reduce stress that arises in the implant bone by lowering the implant occlusion area to the same size as that of the periodontal membrane (approximately 25 μm). Thus, considering that the implant, unlike natural teeth, does not have a periodontal membrane, reducing implant occlusal contact area to lower than that of natural teeth to prevent pressure displacement can prevent implant overload and increase implant survival rate. Meanwhile, Doi et al. (2006) have measured the occlusal force distribution in implant prostheses using a dental prescale and reported that when there was no initial occlusal contact in the implant region, there was low implant occlusal force and occlusal balance could not be maintained during maximum clenching. Furthermore, it has been reported that implant-protected occlusion can cause onset of temporomandibular joint arthrosis (Inai 1998).

We investigated the effects of different occlusal contact sizes of implants on adjacent teeth and demonstrated that when the implant occlusal contact area was smaller than that of adjacent teeth, these teeth exhibited high thresholds for tactile and pressure sensitivity in the periodontal membrane. The results of this study corroborate the findings of Oki et al. (2003), who have used a weighing device that they invented to demonstrate that continuous load on teeth increases periodontal mechanosensitive threshold. Having the implant occlusal contact area in a lower position increases the occlusal burden on the adjacent teeth, causing chronic load to be applied, which appears to have been the reason for high periodontal mechanosensitive threshold exhibited by the adjacent teeth.

In addition, it has been reported that changes in thresholds for tactile and pressure sensitivity occur in patients who complain of occlusal discomfort (Clark and Simmons 2003; Baba et al. 2005) and that occlusal force control and ability to retain food decrease (Trulsson and Gunne 1998) when periodontal mechanoreceptors are missing. It appears that this is because distortion occurs in the periodontal mechanoreceptor stimulation input system, impairing the signal transduction system from the peripheries to the higher centers and the information processing mechanism of the central nervous system.

Currently, implant survival rate is extremely high and the preservation of remaining teeth and maintenance of occlusal function is garnering attention. Aiming for not only recovery of occlusal function with implant treatment but also the long-term preservation of remaining teeth should result in maintenance of oromandibular function. However, it is not necessarily best to increase the implant region occlusal contact in all cases. This is because, as mentioned above, indiscriminately increasing the burden on implants could cause implant overload. Thus, occlusal balance between the implant and remaining teeth is important.

This study has a limitation. Because it is a cross-sectional study, it is difficult to prove a cause-and-effect relationship between the factors and results. However, results suggest that there is a relationship between implant occlusal contact area and periodontal mechanosensitive threshold. Because the measurement methods used can all be easily replicated in the clinical setting, it will be simple to perform further investigation. In the future, we believe that it will be necessary to investigate complex confounding factors for their effects on the remaining teeth in implant therapy, such as their association with the habitual chewing side and occlusal force.

## Conclusions

The results of this study suggest that in cases where at least 3 years have passed following mandibular free-end edentulous implant treatment, there is a relationship between the implant occlusal contact size and periodontal mechanosensitive threshold of adjacent premolars. In our study, a smaller occlusal contact area resulted in increased thresholds. This suggests that the amount of occlusal contact area allocated around the implant region should be considered for long-term maintenance of oromandibular function, including the preservation of adjacent teeth.
